# Perceived time is spatial frequency dependent

**DOI:** 10.1016/j.visres.2011.03.019

**Published:** 2011-06-01

**Authors:** C. Aaen-Stockdale, J. Hotchkiss, J. Heron, D. Whitaker

**Affiliations:** Bradford School of Optometry & Vision Science, University of Bradford, UK

**Keywords:** Time perception, Spatial frequency, Duration, Oddball, Subjective time

## Abstract

We investigated whether changes in low-level image characteristics, in this case spatial frequency, were capable of generating a well-known expansion in the perceived duration of an infrequent “oddball” stimulus relative to a repeatedly-presented “standard” stimulus. Our standard and oddball stimuli were Gabor patches that differed from each other in spatial frequency by two octaves. All stimuli were equated for visibility. Rather than the expected “subjective time expansion” found in previous studies, we obtained an equal and opposite expansion or contraction of perceived time dependent upon the spatial frequency relationship of the standard and oddball stimulus. Subsequent experiments using equi-visible stimuli reveal that mid-range spatial frequencies (ca. 2 c/deg) are consistently perceived as having longer durations than low (0.5 c/deg) or high (8 c/deg) spatial frequencies, despite having the same physical duration. Rather than forming a fixed proportion of baseline duration, this bias is constant in additive terms and implicates systematic variations in visual persistence across spatial frequency. Our results have implications for the widely cited finding that auditory stimuli are judged to be longer in duration than visual stimuli.

## Highlights

► The perceived duration of visual stimuli varies with spatial frequency content. ► Subjective time expansion in response to oddball stimuli is not a general result. ► Sounds being perceived as longer than visual stimuli is not a general result.

## 1 Introduction

Our perception of event duration appears to be modulated by our recent sensory history. For example, the perceived duration of the first stimulus in a stream of identical stimuli is typically overestimated (Rose & Summers, 1995). A related effect concerns the perception of infrequent or unexpected “oddball” stimuli whose perceived duration is expanded relative to that of expected or frequent “standard” stimuli (Tse, Intriligator, Rivest, & Cavanagh, 2004). It was initially suggested that this “subjective time dilation” increased the perceived duration of oddballs by approximately 30–50% (Tse et al., 2004). However, subsequent studies have suggested that this figure was overestimated (Seifried & Ulrich, 2010), revealing a more modest expansion of around 10% (Chen & Yeh, 2009; Pariyadath & Eagleman, 2007; Ulrich, Nitschke, & Rammsayer, 2006; van Wassenhove, Buonomano, Shimojo, & Shams, 2008). The effect seems to be most robust for stimuli that are expanding in size, i.e. looming or approaching (New & Scholl, 2009; Tse et al., 2004; van Wassenhove et al., 2008), and can be eliminated (New & Scholl, 2009) with contracting or receding oddballs. The effect is reduced (Tse et al., 2004) or reversed (van Wassenhove et al., 2008), when a static oddball is presented within a stream of expanding standards. This implies an ecological “alerting” function and is consistent with reports of time slowing down in threatening situations (Campbell & Bryant, 2007; Stetson, Fiesta, & Eagleman, 2007). Inconsistent with this explanation, however, is the fact that similar effects have been reported for stationary stimuli (Chen & Yeh, 2009; Pariyadath & Eagleman, 2007; Tse et al., 2004).

There are two main competing explanations of subjective time dilation. The arousal theory claims that the alerting effect of an oddball causes a central internal pacemaker (Creelman, 1962; Treisman, 1963) to speed up, resulting in a subjective prolongation of time (Seifried & Ulrich, 2010; Ulrich et al., 2006). This model receives support from the finding that subjective time dilation is a global phenomenon affecting the whole visual field, not just the oddball or its immediate surround (New & Scholl, 2009). The centralised arousal theory is, however, difficult to reconcile with several experimental results: multisensory versions of the subjective time dilation show asymmetric transfer betweens senses (Chen & Yeh, 2009; van Wassenhove et al., 2008); the expansion of perceived duration can be generated with oddballs that are entirely predictable (van Wassenhove et al., 2008) and the fact that supposedly ‘emotive’ stimuli do not result in a greater expansion of perceived time (Pariyadath & Eagleman, 2007).

The information processing theory (Tse et al., 2004), on the other hand, proposes that the rate at which information is processed acts as the pacemaker component of our timekeeping system, in other words, ‘bits’ of information act as a counter with which we estimate the passage of time. This model suggests that the additional processing resources brought to bear when a novel stimulus appears increases the overall rate at which information is processed, and the greater number of bits processed per unit time leads to an expansion of perceived duration. A related model is the “coding efficiency” model (Eagleman & Pariyadath, 2009) where perceived event duration is directly related to the neural resources expended during the event’s processing by the nervous system. In this model, repeated presentations of the expected or ‘standard’ stimulus leads to progressively more efficient encoding of this stimulus – a phenomenon termed *repetition suppression* (Grill-Spector, Henson, & Martin, 2006; Henson & Rugg, 2003) – such that, on re-appearance, reduced neural activity levels induce a perceived *contraction* in the duration of the *standard,* relative to the non-suppressed oddball stimulus. This same mechanism could also explain the “novelty” effect of Rose and Summers (1995).

Although each of these models offer appealing explanations as to why perceived duration is context dependent, a problem common to such models is an inability to explain the criterion adopted by the nervous system when deciding which events should be designated as ‘expected’ or ‘unexpected’. In other words, how ‘odd’ does a stimulus need to be before its perceived duration is deemed to differ from its neighbours? The diverse nature of standard and oddball stimuli deployed makes inferences on this topic somewhat problematic. For example, oddballs have variously been defined by changes in geometric shape (Tse et al., 2004), stimulus size/intensity (New & Scholl, 2009; Seifried & Ulrich, 2010; Ulrich et al., 2006; van Wassenhove et al., 2008), alphanumeric character and photographic image properties (Pariyadath & Eagleman, 2007), often altering multiple stimulus features simultaneously between oddball and standard trials. Although it has recently been proposed that high level factors play a role (Pariyadath & Eagleman, 2007) inferences as to the nature of this role are difficult without precise control over the stimulus parameters in question.

**Fig. 1:**
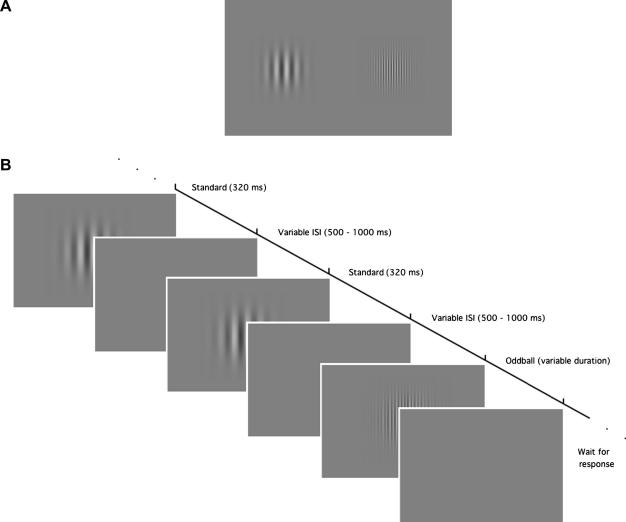
An example of the visual stimuli utilised in all three experiments. The top row of this figure shows two Gabor patches of 2 c/deg (left) and 8 c/deg (right), the values used for the oddball and standard in Experiment 1. The stimuli shown here are of equal physical contrast, whereas in the actual experiments we presented Gabors of equal perceived contrast. The bottom row shows a schematic of a single trial.

In the current study, we presented observers with standard and oddball stimuli that in, phenomenological terms, were obviously different from one another (Fig. 1). However, in a departure from the approaches outlined above, we limited differences between our stimuli to a tightly controlled low-level parameter: spatial frequency. This approach had two advantages. Firstly, it minimises the higher-level cognitive factors that cloud the existing oddball literature. Secondly, it allows the introduction of carefully circumscribed levels of difference in standard and oddball appearance (for example, the difference shown in Fig. 1 reflects a 2 octave change in spatial frequency). Surprisingly, our data show that these low-level stimulus differences fail to induce the classic oddball effect, suggesting that stimulus complexity is perhaps a necessary component of the effect and that other cognitive factors must also be involved. Furthermore, our data show that perceived event duration appears to vary in a systematic fashion with spatial frequency: mid-range frequencies are perceived as longer in duration than high or low frequencies. Our data suggest that this effect forms a constant absolute difference in perceived duration. In other words, the influence of perceived duration appears to be an additive phenomenon which is not tied to the physical duration of the stimulus. This pattern of results is compatible with explanations based around differential levels of visual persistence across different spatial frequency channels.

## 2 General methods

### 2.1 Observers

Six observers took part in Experiment 1 (3 authors plus 3 naives) and 6 took part in Experiment 2 (4 authors plus 2 naives).

### 2.2 Apparatus

All experiments were run on a dual-quad-core Apple Mac Pro computer. Audio and visual stimuli were generated using the Psychophysics Toolbox 3 (http://psychtoolbox.org/) running in a Matlab 7.9 environment and presented either on a Compaq P1220 CRT monitor or via Sennheiser HD 280 headphones. Screen resolution was 1280 × 1024 and refresh rate on the monitor was 100 Hz, giving a frame duration of approx. 10 ms. Stimuli were viewed from 57 cm and a headrest was used to ensure this.

### 2.3 Stimuli

The visual stimuli in all experiments were Gabor patches composed of a sinusoidal grating carrier multiplied by a spatial Gaussian envelope presented on a mean luminance background (Fig. 1). The Gabor patches were all presented in the centre of the screen. The grating component of the Gabor was of varying spatial frequency, but the envelope had a constant standard deviation of 2.7°. Therefore the size of the stimulus was the same for all conditions. The auditory stimulus was a burst of white noise presented binaurally via headphones. The intensity of the auditory stimulus was 65 dB. On- and offset profiles of both the visual and auditory stimuli were rectangular.

## 3 Experiment 1

### 3.1 Methods

In this experiment we carried out a standard oddball task based on previous experiments in the literature. The “standard” stimulus was a 2 or 8 c/deg Gabor patch a series of which were presented for 320 ms to the observer separated by a blank screen for a variable inter-stimulus interval. The observer was initially presented with 10 standard stimuli in order to build up an internal representation of the standard duration. Following this initial phase, each trial consisted of a minimum of 3 and a maximum of 10 presentations of the standard stimulus followed by an “oddball” which was the opposite frequency (2 or 8 c/deg) to the standard. The duration of the oddball was varied symmetrically (Seifried & Ulrich, 2010) around the standard duration in seven steps of 20 ms (oddball durations were 260, 280, 300, 320, 340, 360 and 380 ms) The observer then had to report (via a keypress) whether the oddball appeared longer or shorter than the standard. In between presentations of the standard or oddball stimulus, the screen was mean luminance grey for a variable inter-stimulus interval (isi) of between 500 and 1000 ms. All stimuli (standards and oddballs) were presented at the same spatial location, the centre of the screen and the phase of the sinusoidal component of the Gabor patch was varied randomly on every presentation. Each oddball duration was presented 5 times in each experimental block. Blocks were repeated 4 times to give 20 observations per point. Based upon previous literature, it was hypothesised that we would obtain an expansion of subjective time in both conditions (i.e. an extension of the oddball’s perceived duration), and that this expansion of perceived duration would be roughly equal, on the basis that the spatial frequency of the oddball in both conditions differed from that of the standard Gabor by the same number of octaves.

### 3.2 Equating for visibility

It has been suggested that perceived duration is systematically biased by the contrast or luminance of a stimulus with the most common finding being that brighter (or higher contrast) stimuli are perceived as longer in duration (e.g. Terao, Watanabe, Yagi, and Nishida (2008)). Previous oddball-based studies using images or complex geometric stimuli have not equated for such low-level image characteristics. In order to investigate the effect of stimulus novelty under conditions of matched stimulus visibility, we conducted an initial experiment where we controlled for differences in perceived contrast at different spatial frequencies. We therefore set the low spatial frequency (2 c/deg) Gabor to 50% contrast and asked our observers to match the perceived contrast of the high spatial frequency (8 c/deg) Gabor to this value. We used a temporal 2AFC task in which the observer was presented with either the 2 c/deg Gabor followed by the 8 c/deg Gabor (or vice versa) at the centre of the screen and had to report which interval contained the higher contrast grating. The contrast of the 8 c/deg Gabor was determined by a Quest staircase (Watson & Pelli, 1983) to match its perceived contrast to the 2 c/deg stimulus. Three separate Quest staircases were run and the mean taken. The appropriate value for each observer was then used in Experiment 1. We adopted this contrast matching paradigm because the stimuli used in this experiment were highly suprathreshold. Due to contrast constancy in the visual system, presenting stimuli at very large multiples of threshold may not result in equally “visible” stimuli (Georgeson & Sullivan, 1975).

### 3.3 Results

**Fig. 2:**
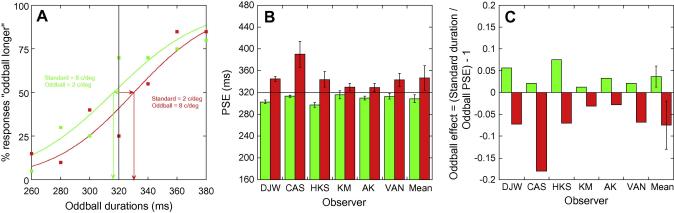
(A) Example psychometric functions for one observer. The green curve shows data from the condition in which the standard Gabor patch is 8 c/deg and the oddball Gabor patch is 2 c/deg. The red curve shows data from the condition in which the standard is 2 c/deg and the oddball is 8 c/deg. Arrows show the shift in the psychometric functions from veridical and the corresponding PSEs. The middle plot (B) shows PSE data for all observers (colour-coding identical to A). Error bars for individuals show the error of the PSE extracted from the logistic function fit to the data. Error bars for the group show the standard deviation. The right-hand plot (C) shows that temporal expansion occurs when the oddball is lower in spatial frequency than the standard (green), while temporal contraction occurs when the oddball is a higher spatial frequency than the standard (red). The direction of the effect is modulated according to the spatial frequency relationship of the stimuli, not their ‘differentness’.

A logistic function of the form

y=100/(1+exp-((x-μ))/θ)

was fitted to the raw data for each observer at each baseline duration, from which the position of subjective equality (PSE – *μ* in the above equation) – the physical oddball duration that was perceived to match the standard duration – was extracted. Samples of the resultant psychometric functions from one observer are shown in the left-hand plot of Fig. 2A. Surprisingly, we obtain equal and opposite shifts from veridical depending upon the spatial frequency relationship of the standard to the oddball, rather than the expected subjective expansion. With a standard of 8 c/deg and an oddball of 2 c/deg (green curve), we see a decrease in the PSE values, signifying an expansion in the perceived duration of the oddball, consistent with previous reports. However, when the stimuli are reversed (red curve) we see an increase in PSE values, signifying a *contraction* of the perceived duration of the oddball. PSE values for each observer and the mean PSE for the two conditions are summarised in Fig. 2B, with bars colour-coded as in 2A. For all observers, when the oddball is lower in spatial frequency than the standard (green bars), there is a decrease in the PSE relative to veridical. Similarly, for all observers, when the oddball is *higher* in spatial frequency than the standard (red bars), there is an *increase* in the PSE from veridical. In order to show this differential effect more clearly we calculated a modified version of the *temporal expansion factor* (TE) used in previous studies (Tse et al., 2004). The TE is the standard duration divided by the oddball PSE. We calculated this value and subtracted it from 1, which gave us a positive value for temporal expansions and a negative value for temporal contractions. This analysis can be seen in Fig. 2C. Notice that all green bars are positive and all red bars are negative.

A paired-samples *t-*test showed that the difference between the perceived duration of the oddball in the different conditions was significant (*t*(5) = −4.1, *p* = <0.05, *r* = 0.1), but the small sample size resulted in a significantly non-normal distribution of scores for the condition in which the oddball was of a higher spatial frequency (Kolmogrov-Smirnov, *D*(6) = 0.372, *p* = <0.05). In light of this non-normality a Wilcoxon rank-sum test was also carried out which also resulted in a significant difference between the conditions (*z* = 2.201, *p* < 0.05). There was no systematic difference in the slope of the psychometric function, therefore no systematic difference in discrimination, between the two conditions (*t*(5) = 1.393, *p* = >0.05, *r* = −0.236).

Given that both oddballs shared matched differences in spatial frequency – relative to their respective standard stimuli – it seems unlikely that the expansion shown for the 2 c/deg oddball condition and the contraction shown in the 8 c/deg oddball condition result from separate mechanisms. Rather, the data in Fig. 2 suggest that the spatial frequency *per se* may be the dominant factor governing the perceived duration of the stimuli employed in Experiment 1. In Experiment 2, we removed issues surrounding stimulus expectancy and investigated perceived temporal extent as a function of spatial frequency.

## 4 Experiment 2

### 4.1 Methods

On each trial, we presented an auditory burst of white noise as a “standard”, after which a Gabor patch with a spatial frequency of 0.5, 1, 2, 4 or 8 c/deg was presented. The spatial frequency of the Gabor patch was randomly interleaved within a block according to the method of constant stimuli. Observers were then asked to make two alternative forced choice duration discrimination judgments as to ‘which was longer, the visual or auditory stimulus?’ and responded via a keypress. The sound was always presented first.

Any differences in perceived duration that we obtained in Experiment 1 could be the result of either differential visual persistence of the stimuli or caused by an inherent biases in the temporal processing of different spatial frequencies. These two different explanations result in two very different predictions. A persistence effect would manifest itself as a constant *additive* difference in perceived duration, which would be proportionally smaller as stimulus duration increased (equivalent to a perceptual increment added to the perceived duration of a particular spatial frequency, which would be constant across different physical durations). On the other hand, a ‘faster’ or ‘slower’ clock for different spatial frequencies would result in an effect that was a constant *proportion* of stimulus duration.

In order to test for this, our auditory standard was centred on a baseline duration of 160, 320 or 640 ms, but the trial-to-trial duration of the auditory standard was jittered by +/−20% around each of these baseline values. This ensured that the observer attended to the auditory standard and could not simply opt to ignore the duration of the noise and compare the duration of the visual stimulus to an internally generated standard (the method of single stimuli). Thus, the exact duration of the standard varied slightly within a block whereas the average baseline duration was varied between blocks.

The duration of the Gabor patch stimulus was chosen from among seven durations that were equally spaced around, and centred on, the standard duration *for that trial.* Logistic functions were then fitted to this raw data for each observer, spatial frequency and baseline duration. From these psychometric functions, the position of subjective equality (PSE) was extracted in the same fashion as previously described for Experiment 1. Since the raw data were expressed as percentages of the standard duration we multiplied the PSEs by the relevant three baseline durations (160, 320 and 640 ms), so that we could express all the data in millisecond terms.

### 4.2 Equating for visibility

For each baseline duration (160, 320 and 640 ms), the perceived contrast of the 0.5, 1, 4 and 8 c/deg Gabor patches was equated to that of a 50% contrast, 2 c/deg Gabor of the appropriate duration using a 2AFC task and interleaved Quest staircases (Watson & Pelli, 1983). The appropriate values for each observer were then used in Experiment 2. Three separate Quest staircases were run and the mean taken.

### 4.3 Results

**Fig. 3:**
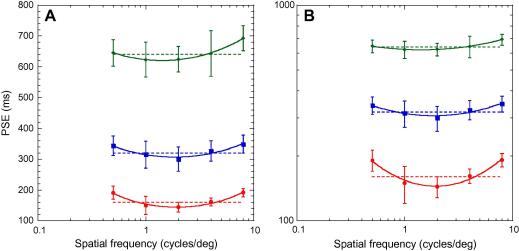
Individual PSEs for five different spatial frequencies and three different baseline durations of 160 (red), 320 (blue) and 640 (green) ms. Group data is presented on a linear (A) and log (B) scale. Dotted lines show the veridical duration of the auditory standard. Error bars show the standard deviation of the group mean.

The average PSEs for seven observers are shown in Fig. 3A and B. The data are fitted with a Gaussian function of the form

$$y=h/e^{-(\log{\left(x/f\right)}^{2}/2\sigma^{2})}$$

where *f* is the spatial frequency corresponding to the minimum of the curve, *h* is the duration in milliseconds at the minimum point of the function and *σ* is the standard deviation.

Fig. 3A shows a clear effect of spatial frequency on perceived duration. The ‘u-shaped’ distribution of these data shows that, relative to the higher and lower ends of the spatial frequency range, middle spatial frequencies are perceived as having a longer duration. This effect appears maximal at around 2 c/deg, and, the effect appears to be constant, in millisecond terms, as baseline duration increases. This can be seen if we change the *y*-axis from a linear scale (Fig. 3A) to a logarithmic scale (Fig. 3B). Plotted like this, the functions are progressively shallower at longer durations, reflecting an effect of spatial frequency that gets proportionally smaller as baseline duration increases. These effects were confirmed by a two-way repeated measures analysis of variance, which revealed that the effect of both baseline duration (*F*_2,10_ = 936.9, *p* < 0.001) and spatial frequency (*F*_4,20_ = 16.17, *p* < 0.001) were highly significant. Importantly, however, there was no significant interaction between these two parameters (*F*_8,40_ = 0.578, *p* > 0.1) indicating that, in absolute terms, the spatial frequency effect was similar across baseline durations.

The spatial frequency dependence of duration perception found in Experiment 2 appears to peak between 1.4 and 2 c/deg (*f* = 1.94 for 160 ms, 1.86 for 320 ms and 1.42 for 640 ms). The minimum of the curve is consistently 10–20 ms lower than the veridical duration (*h* = 145 for 160 ms, 307 for 320 ms and 620 for 640 ms), showing that mid-range spatial frequencies are perceived as *longer* than the auditory standard. Around 1 or 4 c/deg performance is veridical, while at the extremes perceived duration is 20–50 ms *shorter* than veridical.

## 5 Discussion

Our results demonstrate two key findings: Firstly, oddball-related temporal expansion cannot be solely attributable to perceived differences between standard and oddball stimuli: when high level content is minimised, yet (grossly supra-threshold) low-level differences persist, we failed to reproduce the temporal expansion found elsewhere in the literature. Secondly, our data clearly show a significant bias towards perceiving mid-range spatial frequencies (∼2 c/deg) as longer in duration than high (∼8 c/deg) or low (∼0.5 c/deg) frequencies. The effect occurs when visual stimuli of different spatial frequency are compared to each other (Experiment 1) or are compared cross-modally to an auditory standard (Experiment 2).

Within the time perception literature a widely reproduced finding is that sounds are typically perceived as being longer than lights, despite having the same physical duration (Behar & Bevan, 1961; Goldstone, 1968; Goldstone & Lhamon, 1974; Walker & Scott, 1981; Wearden, Edwards, Fakhri, & Percival, 1998). Our data suggest that this bias is dependent upon the frequency content of the visual stimulus. At mid-range spatial frequencies, the perceived duration of the visual stimuli actually *exceeded* the duration of the auditory standard. However, it is noteworthy that previous work comparing the perceived duration of sounds and lights invariably used uniform geometrical stimuli whose spectral content would have been dominated by lower spatial frequencies. The finding that these visual stimuli are perceived as shorter than sounds is, in fact, consistent with our data for low spatial frequencies. If we consider the left-hand portion of the curves shown in Fig. 3, it can be seen that, for this particular choice of visual stimuli, sounds will indeed be perceived as being longer than lights (as shown by the vertical elevation of the data relative to the horizontal dashed lines). What our data highlights, however, is that such a finding is not a universal one, but depends critically upon the spatial frequency content of the visual stimulus.

Hughes, Lishman, and Parker (1992) investigated the perceived duration of images after low- or band-pass spatial filtering. They found that images with a broader spatial frequency content were perceived as being longer in duration than those images with a narrower range of frequencies, regardless of whether the images contained predominantly low or high frequencies. Since widening the pass-band of the filters used to create the stimuli would necessarily cause more mid-range spatial frequencies to be included in the final image, this finding could be explained by the spatial frequency dependence we have demonstrated in this study and may not necessarily be an effect of broader spatial frequency spectra.

Kaneko and Murakami (2009) systematically varied the spatial frequency of grating stimuli in order to investigate whether perceived duration was dependent upon the temporal frequency or the speed of a stimulus. They concluded that perceived duration increased with speed, but since they compared the duration of their moving gratings to a static comparison of the same spatial frequency, they essentially factored out any effect of spatial frequency *per se* on perceived duration.

Several studies in the visual persistence literature have investigated the effect of spatial frequency on the persistence of very short (<100 ms) duration visual stimuli. Over a similar range of spatial frequencies to ours, these studies have found that perceived duration of the stimulus increases monotonically with spatial frequency (Long & Sakitt, 1981; Meyer & Maguire, 1977). The persistence of the *afterimage,* on the other hand, either decreases with increasing spatial frequency (Long & Beaton, 1980; Long & Sakitt, 1981) or is band-pass, depending on mean luminance (Ueno, 1983). Methodological issues and problems of definition have clouded the persistence literature (Bowling & Lovegrove, 1982; Nisly & Wasserman, 1989) and the emphasis on persistence beyond stimulus offset has neglected factors influencing stimulus *onset.* Baro, Brzezicki, Lehmkuhle, and Hughes (1992) demonstrated that reaction times to stimulus offset increased as spatial frequency increased, echoing previous findings. However, they also demonstrated that reaction times to stimulus *onset* increased with spatial frequency at the same rate, implying a constant perceived duration across spatial frequency. Finally, in all of these previous studies, no attempts were made to control for the visibility of the different spatial frequencies (Long & Sakitt, 1981), which makes comparison to our data rather difficult.

We did not vary the envelope size of our Gabor stimuli and since the size of receptive fields scales with spatial frequency (De Valois, Albrecht, & Thorell, 1982), more individual detectors would be activated by the high spatial frequency stimuli than the low spatial frequency stimuli. The greater number of detectors, and therefore greater neural energy expenditure, could contribute to the greater perceived duration. However, this would predict a linear increase, not the u-shaped function we obtain. Future work could utilise stimuli of constant bandwidth to investigate this issue.

Biases in temporal processing may be explained by an internal ‘clock’ running faster or slower, or a greater accumulation of missed ‘ticks’ (Brown, 1997). Another possibility is that – given accumulated experience about the probability of commonly encountered durations – observers may have prior assumptions about the durations of certain stimuli, just as we have a tendency to impose shading patterns consistent with the ‘light from above’ prior (Sun & Perona, 1998). The *perception* of mid-range spatial frequencies being longer in duration may then conceivably reflect a higher incidence of longer *physical* durations for images dominated by these frequencies. However, biases in both clock-based and Bayesian mechanisms would be manifest as a constant proportional bias (Jazayeri & Shadlen, 2010), and with respect to the Bayesian explanation, it is unclear whether certain spatial frequencies would be physically present longer than others in natural vision. The bias in perceived duration that we find across spatial frequency is constant in millisecond terms, and therefore appears to reflect greater persistence for mid-range spatial frequencies. It is tempting to implicate low-level factors such as intensity or contrast sensitivity to explain this bias in stimulus persistence, but by equating the visibility of our stimuli on an observer-by-observer basis, these considerations are unlikely to form a convincing explanation for our effects.

It is noteworthy that we find no evidence of any consistent “subjective time dilation” in response to oddballs. The expansion and contraction effects we obtain in Experiment 1 appear to be approximately equal and opposite and seem to be explained entirely by the difference in perceived duration across spatial frequency observed in Experiment 2. Although we used stimuli equated for visibility, the standard was repeatedly presented and the oddball only infrequently presented, which may have resulted in some contrast adaptation in the spatial frequency channels tuned to the standard. Differences in contrast or intensity may lead to biases in perceived duration; the most common finding being that higher contrast/intensity is perceived as longer. However, if a consistent expansion in the perceived duration of oddballs – as a result of arousal (e.g. Ulrich et al., 2006), information processing (e.g. Tse et al., 2004), repetition suppression (e.g. Eagleman & Pariyadath, 2009) or contrast adaptation – existed over and above the spatial frequency differences noted here, we would expect to see an overall reduction for *all* oddball PSE values shown in Fig. 2A and B. The extent of this reduction would be then modulated via changes in spatial frequency. We do not see this. In addition, the differences in perceived duration we observe are also much smaller in magnitude (∼5% in either direction) than that usually reported for oddball studies. The primary difference between the current study and previous studies is the nature of the stimulus, which in our study is a narrowband, low-level stimulus. Previous studies have variously used dynamic, broadband or cognitively-engaging natural images and it may be that our stimuli, chosen to selectively target low-level visual mechanisms are not “high-level” enough to evoke subjective time dilation. This suggests that “subjective time dilation” effects are essentially high-level in nature, with a neural locus beyond V1, and necessitate the use of complex, dynamic or cognitively-engaging stimuli.

Having demonstrated a persistent bias in the perceived duration of equi-visible gratings of different spatial frequency, it is tempting to contemplate the perceived duration of a compound grating or plaid stimulus composed of multiple frequency components. Would perceived duration be computed in a winner-take-all fashion, with the longest (or shortest) duration dominating, or is perceived duration the mean of the different component durations? Experiments are currently underway to determine this.
